# Exercise training improves obesity-induced inflammatory signaling in rat brown adipose tissue

**DOI:** 10.1016/j.bbrep.2022.101398

**Published:** 2022-11-28

**Authors:** Takamasa Tsuzuki, Toshinori Yoshihara, Noriko Ichinoseki-Sekine, Hiroyuki Kobayashi, Takayuki Negishi, Kazunori Yukawa, Hisashi Naito

**Affiliations:** a Faculty of Pharmacy, Meijo University, Aichi, Japan; b Graduate School of Health & Sports Science, Juntendo University, Chiba, Japan; c Faculty of Liberal Arts, The Open University of Japan, Chiba, Japan; d Department of General Medicine, Mito Medical Center, Tsukuba University Hospital, Ibaraki, Japan

**Keywords:** Brown adipose tissue, Exercise, Inflammation, Uncoupling proteins, Oxidative stress

## Abstract

Chronic inflammation is considered as an etiology of obesity and type 2 diabetes. Brown adipose tissue (BAT) of obese animals shows increased inflammation. Regular exercise has anti-inflammatory effects; however, the effects of exercise training on BAT inflammation in obese animals remain unclear. Thus, this study aimed to investigate the effects of exercise training on inflammation-related signaling in the BAT of obese and diabetic rats. Male Otsuka Long-Evans Tokushima Fatty (OLETF) rats, an obese/diabetic rodent model, were randomly divided into either sedentary (*n* = 11) or exercise training (*n* = 8) groups. Long-Evans Tokushima Otsuka (LETO; *n* = 9) rats were used as the nondiabetic sedentary controls. Exercise training using a treadmill was conducted 4 days per week for 20 weeks, starting at 5 weeks old. As a result, exercise training attenuated the phosphorylation levels of p65 and mitogen-activated protein kinases in the BAT of OLETF rats, concurrently with the improvement of obesity and systemic glucose tolerance. Moreover, exercise training decreased oxidative stress and increased the antioxidant and anti-inflammatory protein levels in the BAT. Conversely, exercise training did not alter the expression levels of uncoupling protein-1 and oxidative phosphorylation-related proteins in the BAT, which were lower in the OLETF rats than the LETO rats. In conclusion, our data suggest that exercise training prevents the activation of inflammatory signaling in the BAT of obese/diabetic rats.

## Highlights

• Exercise training ameliorates the activation of inflammation-related signaling cascades in the BAT of obese rats.
• Exercise training decreased oxidative stress measured by the expression level of protein carbonyl in the BAT of obese rats.
• Exercise training increased the expression levels of antioxidant and anti-inflammatory protein in the BAT of obese rats.

## 1 Introduction

The prevalence of obesity has dramatically increased over the past several decades worldwide [1]. Obesity results from an imbalance between energy intake and expenditure, such as increased consumption of high-calorie and high-fat diets and decreased physical activity. Obesity represents an important risk factor for non-alcoholic fatty liver disease, type 2 diabetes, and cardiovascular disease [2,3]. Thus, there is an imperative need for effective therapies to prevent obesity and its related comorbidities.

Adipose tissues play a crucial role in energy metabolism. Mammalians have two main types of adipose tissues: the white adipose tissue (WAT) and the brown adipose tissue (BAT). WAT is composed of unilocular white adipocytes and functions as energy storage in the form of triglycerides, which enhance the release of hormones and cytokines that regulate whole-body metabolism and insulin resistance [[4], [5], [6]]. Conversely, BAT is characterized by multilocular lipid droplets and abundant mitochondria containing the uncoupling protein-1 (UCP1) [7]. UCP1 uncouples mitochondrial respiration from ATP synthesis, resulting in heat production primarily via non-shivering thermogenesis [8]. To generate heat, BAT utilizes glucose and fatty acids as fuel. Thus, BAT exerts beneficial metabolic effects in combating obesity and metabolic diseases [9].

Obesity is associated with low-grade chronic inflammation of peripheral organs, such as the liver and WAT, which further contributes to systemic inflammation [10]. Particularly, inflammation in WAT is characterized by elevated pro-inflammatory cytokine secretion and infiltration of immune cells, which subsequently activates the inflammatory signaling, including the nuclear factor-κB (NF-κB) and mitogen-activated protein kinases [MAPKs: extracellular signal-regulated kinase 1/2 (ERK1/2), p38, and c-Jun N-terminal kinase (JNK)] [11,12]. Consequently, inflammation is thought to contribute to obesity-related metabolic diseases, such as insulin resistance and type 2 diabetes [13]. Recently, it has been reported that obesity is attended by an increase in the inflammatory response in BAT [14], and inflammation in BAT suppresses UCP1 expression and mitochondrial respiration [15].

Physical exercise is essential to human health and is an effective means to combat obesity and type 2 diabetes. The beneficial effects of exercise are associated with adaptation to multiple tissues, such as the skeletal muscle, liver, and adipose tissues. Previous studies have identified exercise-induced adaptations to WAT in humans and rodents, including enhanced mitochondrial activity, decreased lipid droplet, improved inflammation, and altered endocrine profiles, which contribute to improved metabolic homeostasis [16,17]. Recently, browning of WAT by exercise has also been investigated in rodent models [18]. However, the effect of exercise on BAT remains conflicting in regard to mitochondrial activity, glucose and lipid metabolism, and thermogenic activity in rodents and humans [19,20]. In particular, little is known about the effects of exercise training on the inflammatory responses in BAT. This study aimed to investigate whether exercise training attenuates obesity-induced upregulation of inflammatory signaling in the BAT of obese rats.

## 2 Materials and methods

### 2.1 Animals

All procedures were approved by the Juntendo University Animal Care and Use Committee and were conducted according to the guiding principles for the Care and Use of Laboratory Animals set forth by the Physiological Society of Japan (H24-1). Four-week-old male Otsuka Long-Evans Tokushima Fatty (OLETF) rats, an obese and type 2 diabetic rat model, and their counterpart Long-Evans Tokushima Otsuka (LETO) rats were purchased from Japan SLC (Shizuoka, Japan). OLETF rats exhibit hyperphagia and obesity beginning during early in life and develop insulin resistance and type 2 diabetes [21]. At five weeks of age, OLETF rats were randomly assigned to a sedentary group (SED, *n* = 11) or an exercise training group (TR, *n* = 8), and LETO rats (*n* = 9) were kept sedentary. The rats were housed in an environment-controlled animal facility (24 ± 1 °C, 55 ± 1%) and illuminated with a 12:12-h light-dark cycle. The animals were provided with standard rodent chow and water *ad libitum*.

### 2.2 Exercise training

The rats in the TR group were trained using a motor-driven animal treadmill (KN-73; Natsume, Tokyo, Japan) 4 days per week for 20 weeks. The exercise was started at Zeitgeber Time (ZT) 16 during the dark (active) period. The exercise speed and duration were gradually increased and attained at 25 m/min for 60 min until 6 weeks of training period. After this period, the exercise speed was further increased until 30 m/min during 7–20 weeks of training period. Electric shocks (1–1.5 mA) were rarely used to motivate the rats to run when the rats stay on an electrode for several seconds. LETO rats and SED group in OLETF rats were handled in the same manner as the TR group and were placed beside the treadmill during exercise training but were not exposed to the electric shock.

### 2.3 Glucose tolerance test

To assess the systemic glucose tolerance, we performed the intraperitoneal glucose tolerance test (GTT) during ZT16-18. Glucose (1.0 g/kg body weight) was intraperitoneally administered to LETO and OLETF rats after overnight fasting at 25 weeks of age. Blood samples were collected immediately before and at 30, 60, and 120 min after glucose injection. Blood glucose levels were measured using the Glutest Neo Super®︎ device (Sanwa Kagaku Kenkyusyo, Nagoya, Aichi, Japan).

### 2.4 Sampling and sample preparation

After the training period, the rats were anesthetized with pentobarbital sodium (60 mg/kg) 48 h after the final bout of the exercise training. Blood samples were collected from an abdominal vein. Interscapular BAT was removed, weighed, frozen in liquid nitrogen, and stored at −80 °C until Western blot analysis.

BAT was homogenized in ice-cold RIPA buffer (25 mM Tris-HCl, pH 7.6, 150 mM NaCl, 1% NP-40, 1% sodium deoxycholate, 0.1% SDS; Thermo Scientific, Wilmington, CA, USA) containing 10 mM EDTA, Halt™ protease inhibitor cocktail EDTA-free (Thermo Scientific), and PhosSTOP (Roche, Penzberg, Germany). The homogenates were centrifuged at 700×*g* for 5 min at 4 °C, and the lipid layer was removed, and the supernatants were centrifuged again at 12,000×*g* for 15 min at 4 °C. Protein concentrations in the supernatant were determined using the BCA Protein Assay Kit (Thermo Scientific). Protein extracts were solubilized in sample buffer (30% glycerol, 5% 2-beta-mercaptoethanol, 2.3% SDS, 62.5 mM Tris-HCl pH 6.8 and 0.05% bromophenol blue) at 2.0 mg/mL and incubated at 95 °C for 5 min.

### 2.5 Immunoblot analysis

Equal amounts of protein were loaded onto 10% sodium dodecyl sulfate polyacrylamide gel electrophoresis (SDS-PAGE) gels and run at 150 V for 50–60 min. Proteins were then transferred to polyvinylidene difluoride (PVDF) membranes at 100 V for 60 min. After transfer, the membranes were blocked for 1 h at room temperature in PVDF blocking reagent (TOYOBO Co. Ltd., Osaka, Japan). After three washes with Tween-Tris-buffered saline (T-TBS; 40 mM Tris-HCl, 300 mM NaCl, and 0.1% Tween 20, pH7.5), the membranes were incubated with the primary antibodies in dilution buffer overnight at 4 °C (Supplementary Table S1). After several washes in T-TBS, the membranes were incubated with anti-rabbit or mouse horseradish peroxidase (HRP)-conjugated secondary antibodies (#7074 or #7076, Cell Signalling Technology) in a dilution buffer for 1 h at room temperature. After several washes, bands were visualized using Immobilon Western Chemiluminescent HRP Substrate (Millipore Corporation, Billerica, MA, USA), and the signals were recorded using Fusion FX (Vilber, Marne-la-Vallee, France). Analyses were performed using the Evolution Capture software (Vilber). Protein phosphorylation was calculated as the ratio of phosphorylated to total protein levels and is expressed as arbitrary units. Immunodetection of β-actin was used as a loading control.

### 2.6 Measurement of protein carbonyl

To determine the levels of protein oxidation, an OxiSelect™ protein carbonyl immunoblot kit (STA-308, Cell Biolabs, Inc., CA, USA) was used, according to the manufacturer's instructions. After SDS-PAGE and transblotting, the membranes were incubated with 2,4-dinitrophenylhydrazine (DNPH) in 2N–HCl for 5 min. After several washes in 2N–HCl, the membranes were blocked for 1 h at room temperature in PVDF blocking reagent (TOYOBO). Membranes were incubated overnight with an anti-dinitrophenylhydrazine (DNP) primary antibody at 4 °C. The membranes were incubated with the HRP-conjugated secondary antibody, and DNP signals were visualized using Immobilon Western Chemiluminescent HRP Substrate (Millipore Corporation). To quantify the amount of protein oxidation, the ratio between the densitometric values of carbonyl proteins and those stained with Ponceau S (Beacle, Inc., Kyoto, Japan) was calculated.

### 2.7 Measurement of plasma insulin concentration

Blood samples were centrifuged at 3000 rpm for 10 min to obtain plasma. Insulin concentration was measured using a commercially available enzyme-linked immunosorbent assay kit (Morinaga Institute of Biological Science, Inc., Kanagawa, Japan), according to the manufacturer's instructions.

### 2.8 Statistical analysis

The data are expressed as the mean ± standard error. Statistical significance was determined using one-way or two-way analysis of variance (ANOVA) with Bonferroni's post-hoc test. Statistical significance was set at *p* < 0.05. All statistical analyses were performed using the Prism software ver. 8.1.1. (GraphPad Software, La Jolla, CA, USA).

## 3 Results

### 3.1 Body weight, BAT mass, and systemic glucose tolerance

**Fig. 1:**
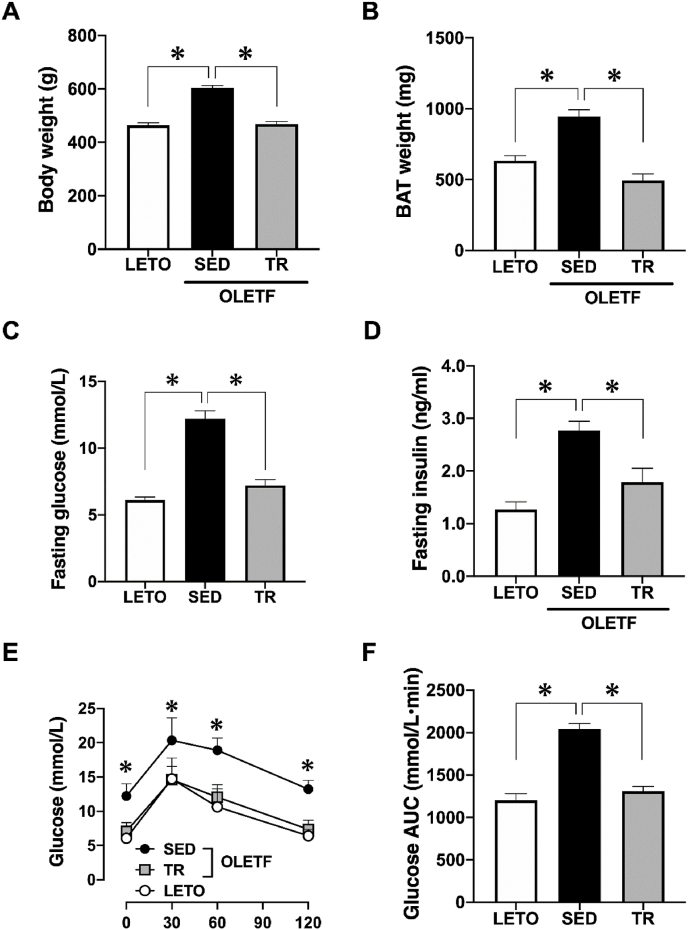
Exercise training prevented obesity and systemic insulin resistance in the OLETF rats Body weight (A) and the weight of brown adipose tissue (B) were recorded at the end of the experiment. Fasting glucose (C) and insulin (D) levels were measured after overnight fasting. We performed a glucose tolerance test (E), and the area under the curve was calculated (F). Long-Evans Tokushima Otsuka (LETO), *n* = 9; sedentary group (SED), *n* = 11; exercise training group (TR), *n* = 8. Values are expressed as mean ± standard error (SE). *p < 0.05.

Body weight and BAT mass of sedentary OLETF rats were significantly greater than those of LETO rats, while exercise training significantly decreased the body weight and BAT mass (*p* < 0.05, Fig. 1A and B). Similarly, fasting glucose and insulin concentrations in sedentary OLETF rats were higher than those in LETO rats, while exercise training significantly decreased these concentrations in OLETF rats (*p* < 0.05, Fig. 1C and D). In addition, GTT confirmed that glucose tolerance was impaired in sedentary OLETF rats and was subsequently normalized by exercise training (*p* < 0.05, Fig. 1E and F).

### 3.2 Phosphorylation of inflammatory related signaling

**Fig. 2:**
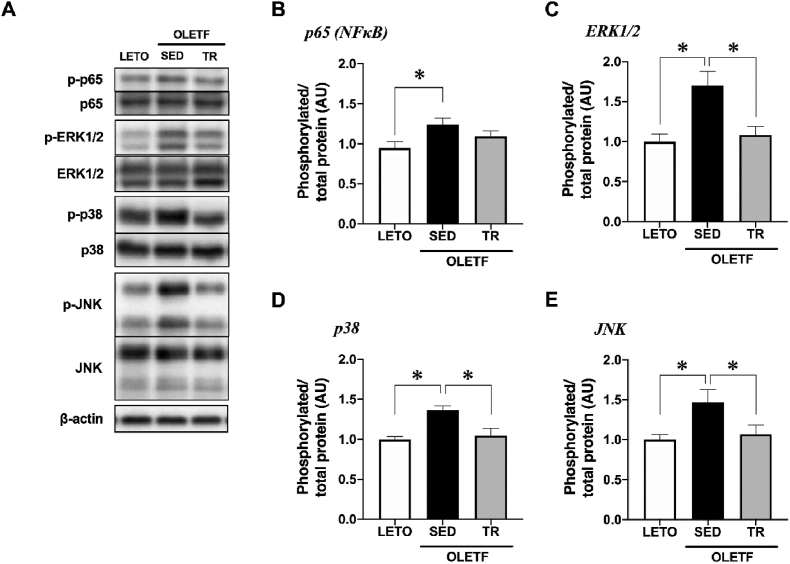
Exercise training attenuated the phosphorylation of inflammatory related signaling in the BAT Representative blots (A) and phosphorylation levels of p65 (B), ERK1/2 (C), p38 (D), and JNK (E) were analyzed by Western blot technique. Long-Evans Tokushima Otsuka (LETO), *n* = 9; sedentary group (SED), *n* = 11; exercise training group (TR), *n* = 8. Values are expressed as mean ± standard error (SE). *p < 0.05.

The phosphorylation level of p65, a subunit of NF-κB, was significantly increased in the BAT of sedentary OLETF rats compared to LETO rats (*p* < 0.05, Fig. 2B). In addition, the phosphorylation levels of MAPKs (ERK1/2, p38, and JNK) were significantly increased in the BAT of sedentary OLETF rats compared with those observed in LETO rats, while exercise training attenuated the increase in the phosphorylation levels of MAPK (*p* < 0.05, Fig. 2C−E).

### 3.3 Expression of oxidative stress markers

**Fig. 3:**
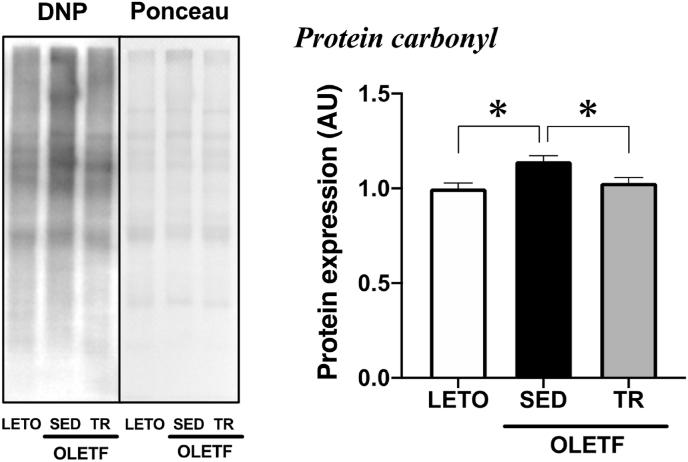
Exercise training decreased obesity-induced oxidative stress in the BAT Representative blot and protein expression level of protein carbonyl, a marker of oxidative stress, were analyzed by Western blot technique. Long-Evans Tokushima Otsuka (LETO), *n* = 9; sedentary group (SED), *n* = 11; exercise training group (TR), *n* = 8. Values are expressed as mean ± standard error (SE). *p < 0.05.

The expression level of protein carbonyl, a marker of protein oxidation, was significantly increased in the BAT of sedentary OLETF rats compared to LETO rats, while exercise training attenuated the increase in the expression levels (*p* < 0.05, Fig. 3).

### 3.4 Expression of anti-inflammatory and antioxidant-related proteins

**Fig. 4:**
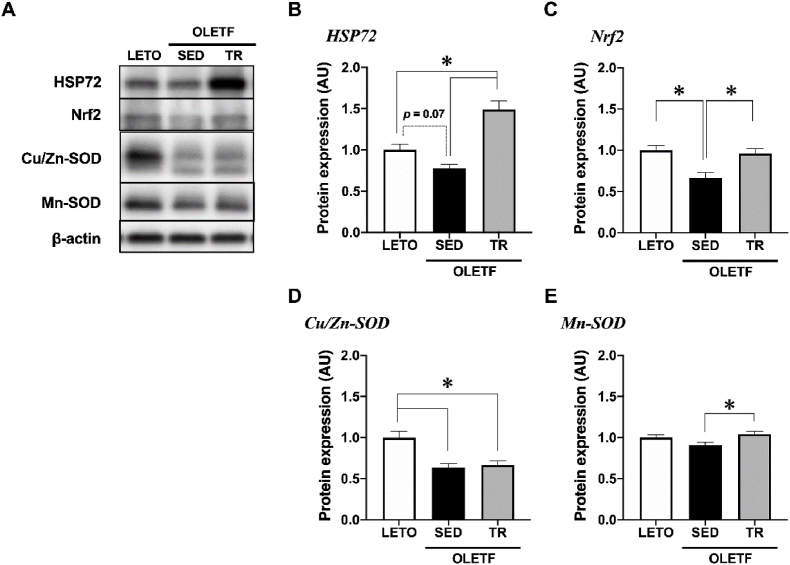
Exercise training upregulated antioxidant-related proteins in the BAT Representative blots (A) and protein expression levels of HSP72 (B), Nrf2 (C), Cu/Zn-SOD (D), and Mn-SOD (E) were analyzed by Western blot technique. Long-Evans Tokushima Otsuka (LETO), *n* = 9; sedentary group (SED), *n* = 11; exercise training group (TR), *n* = 8. Values are expressed as mean ± standard error (SE). *p < 0.05.

The expression level of heat shock protein 72 (HSP72) tended to decrease in the BAT of sedentary OLETF rats compared to that observed in LETO rats (*p* = 0.07), while exercise training significantly increased the HSP72 expression level in OLETF rats (*p* < 0.05, Fig. 4B). The expression level of nuclear factor erythroid 2-related factor 2 (Nrf2) was significantly decreased in the BAT of sedentary OLETF rats compared to that observed in LETO rats, while exercise training significantly increased the Nrf2 expression level in OLETF rats (*p* < 0.05, Fig. 4C).

Moreover, the expression level of Cu/Zn-superoxide dismutase (SOD), a cytosolic antioxidative enzyme, was significantly decreased in the BAT of OLETF rats compared to that observed in LETO rats (*p* < 0.05, Fig. 4D); however, exercise training did not change the Cu/Zn-SOD expression level. On the other hand, the expression level of Mn-SOD, a mitochondrial antioxidative enzyme, was slightly but significantly increased in the BAT of OLETF rats by exercise training (*p* < 0.05, Fig. 4E).

### 3.5 Expression of uncoupling proteins and oxidative phosphorylation-related proteins

**Fig. 5:**
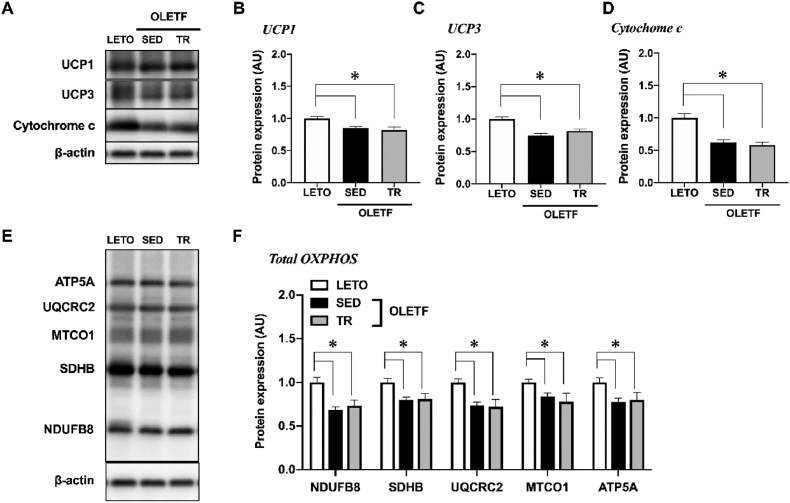
Exercise training did not alter mitochondrial proteins in the BAT Representative blots (A, E) and protein expression levels of UCP1 (B), UCP3 (C), Cytochrome *c* (D), and total OXPHOS (NDUFB8, SDHB, UQCRC2, MTCO1 and ATP5A) (F) were analyzed by Western blot technique. Long-Evans Tokushima Otsuka (LETO), *n* = 9; sedentary group (SED), *n* = 11; exercise training group (TR), *n* = 8. Values are expressed as mean ± standard error (SE). *p < 0.05.

The expression levels of UCP1 and 3 were significantly decreased in the BAT of sedentary OLETF rats (*p* < 0.05, Fig. 5A−C); however, exercise training did not alter these expression levels.

The expression levels of cytochrome *c* and oxidative phosphorylation-related proteins, NDUFB8, SDHB, UQCRC2, MTCO1 and ATP5A, were significantly decreased in the BAT of sedentary OLETF rats compared to those observed in LETO rats (*p* < 0.05, Fig. 5D−F), but these expressions were not changed by exercise training.

## 4 Discussion

Exercise training is a well-established approach to combat obesity and its comorbidities and improves metabolic abnormalities in peripheral tissues, such as the skeletal muscle, liver, and WAT. However, the effects of exercise training on obesity-induced inflammation in BAT remain unknown. Here, we showed that exercise training ameliorates the activation of inflammation-related signaling cascades in the BAT of obese/diabetic rats, concurrently with the improvement of obesity and systemic glucose tolerance. Moreover, exercise training decreased oxidative stress and increased the antioxidant and anti-inflammatory protein levels in the BAT. To the best of our knowledge, this is the first report to show that exercise training improves obesity-induced inflammatory response and oxidative stress in the BAT.

Chronic inflammation of peripheral organs, such as the liver and WAT, is a feature of metabolic diseases, including obesity and type 2 diabetes. Recently, it has been reported that BAT in obese rodent models also exhibits high levels of inflammatory markers, such as pro-inflammatory cytokines and infiltrated immune cells [22,23]. Moreover, activation of receptor-mediated inflammation, such as Toll-like receptors, induces activation of NF-κB and MAPKs signaling in BAT [15]. The activation of these signaling cascades induces an increased inflammatory response, resulting in the development of insulin resistance and other metabolic abnormalities. In the present study, exercise training ameliorated obesity-induced activation of inflammatory signaling cascades, NF-κB and MAPKs, in the BAT, suggesting that exercise training can improve the inflammatory response in BAT. Fat accumulation in peripheral tissues induces the activation of NF-κB and MAPKs, leading to local inflammation. Exercise enhances fatty acid oxidation, resulting in a decreased accumulation of fat in the local tissues. Our data also showed that exercise training decreased the BAT mass in obese rats. In this study, it is assumed that exercise training enhances lipolysis in the BAT and that lipid droplets become smaller, resulting in the improvement of inflammation in the BAT.

Inflammation is often concomitant with oxidative stress, which is a pathophysiological feature of obesity. Since BAT has a very high oxidative capacity, increased energy expenditure is accompanied by the generation of large quantities of reactive oxygen species (ROS). A previous study reported that BAT from obese animals showed a nearly two-fold increase in ROS generation compared to that in lean animals [14]. On the other hand, regular exercise training positively alters the oxidative homeostasis of cells and tissues by decreasing the basal levels of oxidative damage and increasing antioxidant capacity to protect against the harmful effects of oxidative stress [24]. In the present study, protein carbonyl, a marker of oxidative stress, was increased in the BAT of obese rats, whereas exercise training normalized the oxidative stress that was observed in the BAT of obese rats. In addition, the expression of several antioxidant proteins (HSP72, Nrf2 and Mn-SOD) increased in the BAT after exercise training. A previous *in vitro* study reported that HSP72 can modulate stress-activated signaling by directly inhibiting JNK [25]. It is thought that HSP72 elevation induced by exercise training in BAT might also contribute to the suppression of JNK activation, as observed in the present study. Moreover, Nrf2 is an important transcription factor that protects against oxidative stress and increases the expression of antioxidant enzymes. Previous studies have indicated that Nrf2 and NF-κB signaling pathways interact to control the transcription or function of downstream target proteins [26]. NF-κB can directly inhibit Nrf2-antioxidant response element signaling [27], whereas Nrf2 negatively regulates the NF-κB signaling pathway by increasing antioxidant defense [28]. In this study, the increase in Nrf2 expression in the BAT by exercise training, which subsequently leads to an increase in Mn-SOD expression, may be partially involved in the attenuation of oxidative stress and inflammatory signaling in BAT.

Mitochondrial dysfunction is associated with obesity and type 2 diabetes [29]. Additionally, inflammation caused by metabolic disorders negatively regulates UCP1 expression and thermogenic activity in the BAT [30]. Our data showed that the expression levels of oxidative phosphorylation-related proteins and UCPs in the BAT were lower in obese rats than that in non-obese control rats. It is possible that mitochondrial dysfunction induced by metabolic disorders occurred in the BAT as it did in the other tissues. Conversely, the expression levels of these proteins were not altered by exercise training in this study. It suggests the possibility that the BAT activity was not altered by exercise because BAT-specific UCP1 expression did not change. Currently, a randomized controlled trial in young sedentary adults has revealed that 24-week exercise intervention does not affect BAT volume and activity as evaluated by ^18^F-fluorodeoxyglucose uptake into BAT [20]. However, in the rodent studies, the effects of exercise training on mitochondrial content or activity in BAT remain controversial; some studies have suggested that mitochondrial activity increased [[31], [32], [33]], decreased [34], or did not change [[35], [36], [37]] in the BAT with exercise training. BAT thermogenic activity is influenced by the activation of the sympathetic nervous system. The stimulation of cyclic adenosine monophosphate (cAMP) -dependent signaling pathway via β-adrenergic receptor (β-AR) results in heat production by UCP1 [19]. Cold exposure is the most well-studied mechanism of BAT activation via β-AR-cAMP-dependent signaling to generate heat. However, exercise itself is a thermogenic activity; therefore, it might not be necessary to upregulate BAT activity through exercise. Although a recent study has reported that the exercise-induced secreted factors (for example, myokine secreted from skeletal muscle) modulate mitochondrial function and UCP1 expression [38], the effect of exercise on BAT activity remains elusive. Therefore, a further discussion is necessary about the relation between exercise and BAT activity.

Our study has some major limitations. First, we did not measure the systemic inflammatory markers. Some previous studies have reported that exercise training decreases circulating cytokines, such as tumor necrosis factor-α [39]. It is possible that the improvement in the inflammatory response in the BAT might be involved in the decrease in circulating cytokines by exercise training. Second, it is unclear that how much the amelioration of inflammation in BAT can affect improvement of systemic glucose tolerance. Decreasing inflammation leads to the upregulation of insulin sensitivity and subsequently glucose metabolism in the peripheral tissues such as skeletal muscle and liver, resulting in improving systemic glucose metabolism. Although exercise training ameliorated the activation of inflammatory signaling in this study, we did not evaluate the insulin sensitivity or glucose metabolism in BAT. Previous studies in rodents and human have reported that exercise decreases glucose uptake into the BAT [34,40]. These data suggested that it is possible that the primary role of exercise on BAT is not to stimulate glucose uptake into BAT [19]. Although, to date, the effects of exercise on glucose metabolism in BAT have been controversial, the contribution of BAT to the improvement of systemic glucose tolerance by exercise training might be smaller than that of other tissues such as skeletal muscle and liver. Finally, the effect of non-exercise stress, especially electric shocks during exercise training, on the outcomes in this study are uncertain because we did not measure any stress markers such as the weight of adrenal gland or thymus, and blood corticosterone level. In any case, further studies should be conducted considering these limitations.

## 5 Conclusions

Exercise training ameliorates obesity-induced activation of inflammatory signaling in the BAT in obese/diabetic rats, concurrently with the improvement of obesity and systemic glucose tolerance. Further research is needed in the future on how the amelioration of inflammation in the BAT contributes to improving systemic inflammation and metabolism.

## Author contributions

Conceptualization: Takamasa Tsuzuki, Hiroyuki Kobayashi, Hisashi Naito.

Data curation: Takamasa Tsuzuki, Toshinori Yoshihara, Noriko Ichinoseki-Sekine.

Formal analysis: Takamasa Tsuzuki.

Funding acquisition: Takamasa Tsuzuki, Kazunori Yukawa, Hisashi Naito.

Investigation: Takamasa Tsuzuki, Toshinori Yoshihara, Noriko Ichinoseki-Sekine.

Methodology: Takamasa Tsuzuki, Toshinori Yoshihara, Noriko Ichinoseki-Sekine, Takayuki Negishi.

Project administration: Takamasa Tsuzuki.

Supervision: Hisashi Naito.

Validation: Toshinori Yoshihara, Noriko Ichinoseki-Sekine, Hiroyuki Kobayashi, Takayuki Negishi.

Visualization: Takamasa Tsuzuki.

Writing – original draft: Takamasa Tsuzuki, Toshinori Yoshihara, Noriko Ichinoseki-Sekine.

Writing – review & editing: Takamasa Tsuzuki, Toshinori Yoshihara, Noriko Ichinoseki-Sekine, Hiroyuki Kobayashi, Takayuki Negishi, Kazunori Yukawa, Hisashi Naito.

## Funding

This work was supported in part by grants from the MEXT-Supported program for the Strategic Research Foundation at Private Universities (S1101008). The funder had no role in study design; the collection, analysis, and interpretation of data; the writing of the report; and decision to submit the article for publication.

## Declaration of competing interest

The authors declare that they have no known competing financial interests or personal relationships that could have appeared to influence the work reported in this paper.

## Data Availability

Data will be made available on request.
