# Supplementary material for: Exercise training improves obesity-induced inflammatory signaling in rat brown adipose tissue
Source: Biochem Biophys Rep. 2022 Nov 28;32:101398. doi: 10.1016/j.bbrep.2022.101398 (PMC9713272; doi:10.1016/j.bbrep.2022.101398)
Supplement: Multimedia component 1 [file mmc1.docx]

| **Supplementary Table S1. Primary antibodies used in this study** | | |
| --- | --- | --- |
| **Target** | **Campany** | **Catalog number** |
| β-actin | Cell Signaling Technology | #8457 |
| phopho-p65 NFκB | Cell Signaling Technology | #3033 |
| p65 NFκB | Cell Signaling Technology | #8242 |
| phospho-ERK1/2 | Cell Signaling Technology | #4370 |
| ERK1/2 | Cell Signaling Technology | #4695 |
| phospho-p38 | Cell Signaling Technology | #4511 |
| p38 | Cell Signaling Technology | #8690 |
| phospho-JNK | Cell Signaling Technology | #4668 |
| JNK | Cell Signaling Technology | #9252 |
| Nrf2 | Proteintech | 16396-1-AP |
| HSP72 | Enzo | ADI-SPA-810 |
| Cu/Zn-SOD | Enzo | ADI-SOD-100 |
| Mn-SOD | Enzo | ADI-SOD-110 |
| UCP1 | Abcam | ab10983 |
| UCP3 | Thermo | PA1-055 |
| Cytochrome c | Cell Signaling Technology | #4280 |
| Total-OXPHOS | Abcam | ab110413 |
